# The Future of Targeted Gene-Based Treatment Strategies and Biomarkers in Parkinson’s Disease

**DOI:** 10.3390/biom10060912

**Published:** 2020-06-16

**Authors:** Alexia Polissidis, Lilian Petropoulou-Vathi, Modestos Nakos-Bimpos, Hardy J. Rideout

**Affiliations:** Laboratory of Neurodegenerative Diseases, Centre for Clinical, Experimental Surgery & Translational Research, Biomedical Research Foundation of the Academy of Athens, 11527 Athens, Greece; apolissidis@bioacademy.gr (A.P.); epetvathi@bioacademy.gr (L.P.-V.); modestosbio@hotmail.com (M.N.-B.)

**Keywords:** alpha-synuclein, LRRK2, glucocerebrosidase, GBA, biomarker, disease-modifying, Parkinson’s disease

## Abstract

Biomarkers and disease-modifying therapies are both urgent unmet medical needs in the treatment of Parkinson’s disease (PD) and must be developed concurrently because of their interdependent relationship: biomarkers for the early detection of disease (i.e., prior to overt neurodegeneration) are necessary in order for patients to receive maximal therapeutic benefit and vice versa; disease-modifying therapies must become available for patients whose potential for disease diagnosis and prognosis can be predicted with biomarkers. This review provides an overview of the milestones achieved to date in the therapeutic strategy development of disease-modifying therapies and biomarkers for PD, with a focus on the most common and advanced genetically linked targets alpha-synuclein (*SNCA*), leucine-rich repeat kinase-2 (*LRRK2*) and glucocerebrosidase (*GBA1)*. Furthermore, we discuss the convergence of the different pathways and the importance of patient stratification and how these advances may apply more broadly to idiopathic PD. The heterogeneity of PD poses a challenge for therapeutic and biomarker development, however, the one gene- one target approach has brought us closer than ever before to an unprecedented number of clinical trials and biomarker advancements.

## 1. Introduction

Μore than 6 million people worldwide are living with Parkinson’s disease (PD) and it is estimated that this number will double by the year 2040, making PD the fastest growing neurodegenerative disease [1]. Currently, there is neither a clinical biomarker for early detection, diagnosis or prognosis, nor a cure or disease-modifying treatment available for PD. Thus, it becomes evident that biomarkers and disease-modifying therapies are both urgent unmet medical needs in the field and must be developed concurrently because of the paradox: what good are biomarkers for early detection of disease when no disease-modifying therapies are available, or what good are disease-modifying therapies if there are no biomarkers available to detect the disease early enough, i.e., prior to overt neurodegeneration, so patients can have maximal benefit? Another part of the challenge in developing biomarkers and drugs aiming to modify disease is that the pathophysiology of PD is heterogeneous, multifactorial and early stages of the disease have only recently been studied.

PD is a neurodegenerative disorder characterized by the loss of dopaminergic neurons of the substantia nigra pars compacta and cytoplasmic alpha-synuclein (AS)-rich inclusions termed Lewy bodies (LBs). The hallmark motor symptoms of PD are bradykinesia, rigidity, resting tremor, and postural and gait impairment. PD has also a constellation of non-motor symptoms, including depression, anxiety, psychosis, congnitive impairment, autonomic dysfunction, and sleep disorders, which often precede the motor features by more than a decade [2]. Though the etiology of PD is unknown, ample evidence points towards complex interactions of genetic variants with environmental risk factors [3], with the majority of cases being idiopathic and familial cases accounting for approximately 5–10% of all cases. More than 20 disease-causing gene loci have been associated with PD and 90 independent risk-associated variants based on genome-wide association studies and next generation sequencing [4], many of unknown significance, may potentially play a role in idiopathic PD (iPD) [5]. The α-synuclein (AS) gene (*SNCA*) was the first gene to ever be associated with the development of PD in 1997 [6]. Following this discovery, many more genes have been linked to the disease, including parkin (*PARK2*), UCH-L1 (*PARK5*), PINK1 (*PARK6*), DJ-1 (*PARK7*), leucine-rich repeat kinase-2 (*LRRK2; PARK8*), and ATP13A2 (*PARK9*), as well as the risk genes *GBA1*, *VPS35*, *EIF4G1*, and *PARK16*, which have been linked to PD according to family-based linkage analysis. AS and LRRK2 are associated with autosomal dominant PD, while mutations in parkin, PINK1, DJ-1, and ATP13A2 are linked to autosomal recessive PD. LRRK2 mutations are the most common genetic cause of both familial and iPD and the G2019S mutation is the most common, accounting for 5–6% of familial PD and 1–2% of iPD [7]. Heterozygous mutations in the gene encoding β-glucocerebrosidase (*GBA1*) are considered the greatest genetic risk factor for developing PD (5–10% cases) [8,9].

Male sex is associated with higher incidence and prevalence, earlier disease onset, more severe motor symptoms and progression, and more frequent cognitive decline [10]. The interaction between sex and genetics is complex and understudied. Plasma concentrations of AS (see below) are decreased in males with advanced PD, but not females; and this reduction in plasma AS was associated with greater cognitive impairments and sleep disorders [11]. Interestingly, some studies have found a higher prevalence of LRRK2 mutations among women than among men [12]. LRRK2 mutations also show sex- specific phenotypic effects, including milder motor symptoms and higher cognitive function, a lower incidence of REM sleep behavior disorder (RBD) and worse thermoregulation scores in men with LRRK2-G2019S mutation compared to men with iPD, whereas women with LRRK2-G2019S had worse motor complications than women with iPD [13]. In addition, among the patients with LRRK2 PD, women had worse motor complications but better olfaction than men. Our own biomarker study in iPD and G2019S mutation carriers identified several key sex differences with respect to LRRK2 status. First, we have found overall higher expression levels of LRRK2 in PBMCs from males compared to females, and secondly, the elevated in vitro kinase activity was only evident in PBMCs from male subjects [14]. With respect to *GBA1*, there appears to be an increased risk of neuropsychiatric comorbidities, such as anxiety and depression, in men but not women with *GBA1* mutations [15], suggesting a sex–genotype interplay in PD symptom manifestation.

With respect to biomarkers, we presently focus on biofluid studies (i.e., cerebrospinal fluid (CSF), blood cells and urine). However, other methods including neuroimaging and electrophysiology, along with clinical assessment, provide a vital source of diagnostic and prognostic information and are an equally active area of research. Magnetic resonance imaging (MRI) focusing on structural brain differences looks promising in distinguishing between iPD and other parkinsonian phenotypes (e.g., MSA) [16]. By looking at the functional level, changes in circulating oxygen levels may also differentiate iPD from a healthy state [17]. These abnormalities can occur prior to disease onset; hence, they may also serve as a prognostic tool [18]. Positron emission tomography (PET) or single photon emission computed tomography (SPECT) measure differences at the molecular level by looking, among others, at the dopamine transporter (DAT), approved by the European Medicines Agency as a diagnostic tool [17], dopamine release [17] or glucose metabolism [17,19] using radiolabelled compounds that cross the BBB (e.g., 18F-DOPA, 18F-FDG). In addition, transcranial magnetic stimulation (TMS) provides information about cortical function deficits, a feature commonly observed in movement disorders such as PD [20], and electrophysiology can be used for disease monitoring by measuring neuronal activity directly by wave quantification with the aid of scalp electrodes [21]. To what extent these methods can be used reliably as biomarkers is still questionable and, as PD is a multidimensional disease, a combination of multiple approaches may be essential.

**Figure 1:**
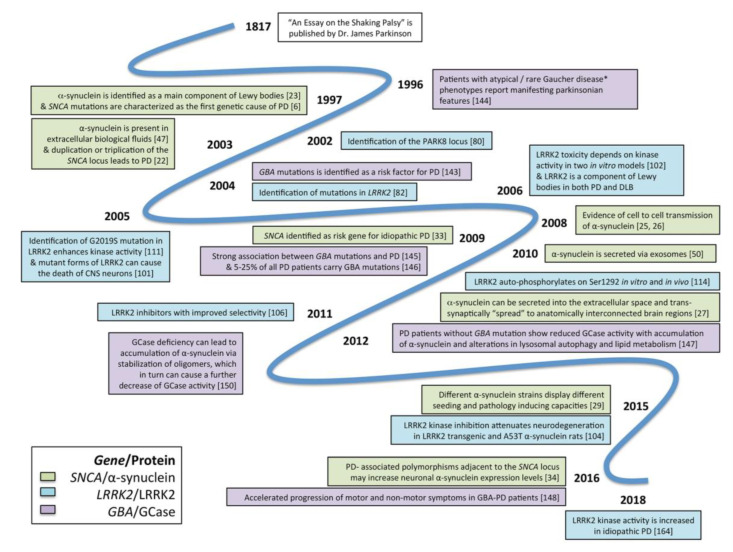
Timeline of milestone discoveries related to the three main genetic PD targets; a-synuclein, LRRK2 and GBA. DLB, Dementia with Lewy bodies; GBA, P-Glucocerebrosidase {Gcase) gene; LRRK2, Leucine—rich repeat kinase 2; PD, Parkinson’s disease; SNCA, α-synuclein gene; * Gaucher disease is a lysosomal storage disorder caused by biallelic GBA mutations.

**Figure 2:**
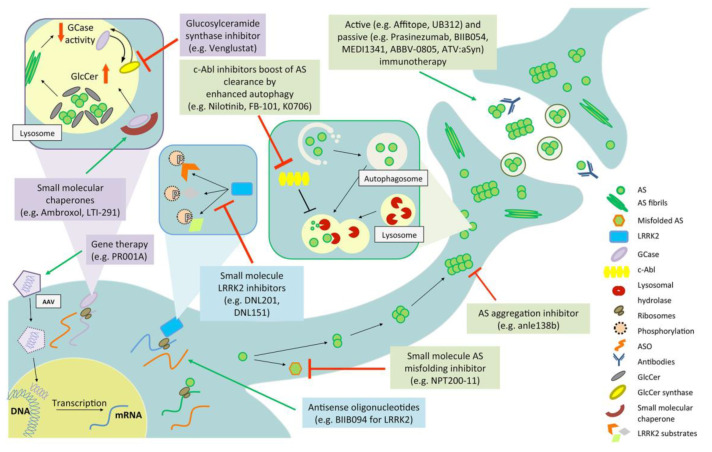
Mechanisms of action of the potential therapeutic compounds currently in clinical trials. As, Alpha-synuclein; ASO, Anti-sense oligonucleotides; GlcCer, Glucosylceramide; LRRK2, Leucine-rich repeat kinase 2.

This review provides an overview of the rationale for and progress in the development of novel biomarkers and disease-modifying treatments for PD. This review focuses on the three most studied genetic pathways—AS, LRRK2 and GBA—that are the most advanced in terms of preclinical proof-of-concept studies, therapeutic development and clinical trial success to date (see Figure 1 for a timeline of milestones, Figure 2 for a summary of drug targets). Finally, we will focus on the three most studied gene targets, alpha-synuclein (AS), glucocerebrosidase (*GBA1*), and leucine-rich repeat kinase-2 (*LRRK2*), but we will also discuss the overlapping commonalities of the three pathways and the importance of patient stratification and how this knowledge pertains to iPD. This is not meant to be an exhaustive literature search but rather to highlight the urgency, bridge the current trends in translational and clinical research in the discovery and development of biomarkers and disease-modifying therapies for PD and, finally, provide the impetus for stimulating, innovative and game-changing research.

## 2. Literature Search Criteria

The literature review encompassing therapeutic strategies targeting these specific proteins used expanded search criteria to include the following:

- “synuclein AND antisense”; “synuclein AND passive/active immunization”.
- “LRRK2 AND kinase AND inhibitor”; “LRRK2 AND antisense”.
- “GBA/GCase/glucocerebrosidase AND therapeutic”; “GBA/GCase/glucocerebrosidase AND ambroxol”.

For the purposes of the literature review with respect to biomarkers, examples of our search terms include the following:

- “synuclein AND CSF; “synuclein AND plasma/serum”; “synuclein AND saliva”.
- “LRRK2 AND CSF”; “LRRK2 AND PBMC/monocyte/neutrophil”; “LRRK2 AND urine”.
- “GBA AND biomarker”; “GCase AND biomarker”; “glucocerebrosidase AND biomarker”; “GBA/GCase/glucocerebrosidase AND activity”.

We used both the PubMed as well as clinicaltrials.gov databases for our review, and did not explicitly exclude publications based on publication date, in order to provide some historical context to the genetic bases of PD pathogenesis, and thus therapeutic discovery.

## 3. Therapies Targeting Alpha-Synuclein

Alpha-synuclein (AS) is a presynaptic protein and arguably has the most central role in PD pathogenesis as: (1) multiplications and mutations of the AS gene (*SNCA*) locus cause familial PD [6,22], (2) aggregated AS is a major constituent of LBs and Lewy neurites, the pathological hallmark in AS-related PD and most idiopathic cases [23] and (3) AS can be secreted into the extracellular space and trans-synaptically “spread” to anatomically interconnected brain regions [24,25,26,27]. In addition, genetic, viral and pathogenic AS-inoculated animal models have demonstrated that overexpression of wildtype, mutant and fibrillary AS in neurons is associated with AS aggregation and nigral neurodegeneration [28], with different AS conformations leading to distinct histopathological and behavioural phenotypes [29]. Finally, polymorphisms that occur within specific regions of the *SNCA* gene increase the overall risk of PD by either increasing the production or slowing the clearance of AS [30,31,32,33,34]. Altogether, AS appears to be linked with both iPD and familial forms of PD. Based on this evidence, several therapeutic strategies currently being pursued include reducing AS load via: (1) reduction in gene and protein expression, (2) restoration of AS proteostasis or (3) reduction in AS propagation. There are currently 10 active clinical studies (www.clinicaltrials.gov), in which AS is the primary drug target (summarized in Table 1).

### 3.1. AS Expression Reduction

α-synuclein (AS) was the first gene (*SNCA)* to ever be associated with the development of PD [6]. Duplication or triplication of the *SNCA* locus leads to PD [22] and a number of missense mutations have been identified, of which A53T is the most common [6]. PD-associated polymorphisms adjacent to the AS gene locus have recently been identified and may possibly increase neuronal AS levels [34], supporting the use of AS-lowering therapies in idiopathic cases as well. Thus, one inevitable approach, following the therapeutic success of antisense oligonuleotides (ASOs) for spinal muscular atrophy [35] and Huntington disease [36], includes the inhibition of AS expression via targeted ASOs. ASOs are single-stranded RNA oligomers that are complimentary to specific sequences within a mRNA molecule. Once bound, the mRNA is digested, typically via RNAse H. This approach is currently being pursued at the preclinical level and has demonstrated sufficient AS dowregulation in rodents and non-human primates (Ionis pharmaceuticals; [37]).

A fascinating epidemological study in Norway regarding the long-term use of β2-adrenoreceptor agonists and antagonists leading to reduced and increased risk of PD, respectively, also experimentally demonstrated that β2 receptors regulate AS transcription [38]. These findings led to hope for repurposing β2-adrenoreceptor agonists as potential disease-modifying treatment of PD, however, more recent epidemiological studies have failed to demonstrate these correlations and consider the relative protection with β2-adrenoreceptor agonists to be an indirect effect of smoking (an established reduced risk factor for PD) and the higher risk with β2-adrenoreceptor antagonists to reverse causation, i.e., PD symptoms trigger their use rather than their use triggering PD [39,40]. Thus, further studies are warranted to explore the potential for pharmacologically modulating AS expression via β2 receptors.

### 3.2. Restoration of AS Proteostasis

The presence of AS aggregates in LB inclusions of diseased brains suggests dysfunctional proteostasis due to numerous factors including gene mutations, post-translational modifications, external toxins, neuroinflammation, oxidative stress and age-related dysfunction of proteolysis [41]. These aberrant forms of AS, especially the soluble oligomeric forms, were identified as the main culprit of cell death [42]. Thus, by restoring proteostasis by either inhibiting protein misfolding and aggregation or enhancing AS clearance, AS pathology could potentially be diminished, possibly slowing or halting disease progression. Based on this evidence, NPT200-11 (UCB0599, UCB/Neuropore Therapies), a small molecule AS misfolding inhibitor has shown promise in improving AS pathology and motor function in vivo [43] and completed phase I trials for tolerability in 2016 (NCT02606682). Anle18b (MODAG GmbH), an AS aggregation inhibitor is currently being developed for the synucleinopathy Multiple System Atrophy, with potential for future use in PD, is now in a Phase 1 trial for safety and tolerability (NCT04208152). Alternatively, glyceryl phenybutyrate, an FDA approved drug for urea cycle disorders, is being tested in a phase I trial (sponsored by the University of Colorado) to assess whether or not it can boost the clearance of AS from the brain into the bloodstream (NCT02046434). Another approach includes the enhancement of autophagic degradation of AS with c-Abl tyrosine kinase inhibitors [44,45]. The anti-cancer drug, nilotinib, an inhibitor of the tyrosine kinase c-Abl, is being tested in phase 2 trials for tolerability, safety, pharmacokinetics and motor symptom improvement (sponsored by Georgetown University, NCT02954978; and Northwestern University, NCT03205488), the first of which demonstrated safety, increased CSF levels of the dopamine metabolite homovanillic acid, and a reduction of oligomeric AS [45]. However, the second study, in press, found no evidence of symptomatic benefit, low CSF drug exposure and no changes in CSF dopamine metabolite levels [46]. Furthermore, K0706 (SCC-138) is being tested for safety and efficacy in a phase 2 trial (Sun Pharma Advanced Research Company, NCT03655236) and FB-101 entered phase I trials last year to test safety, tolerability and pharmacokinetics (1ST Biotherapeutics, NCT04165837).

### 3.3. Trans-Synaptic Transmission Reduction

AS can be secreted into the extracellular space [47,48,49,50], taken up by neighboring neurons, recruiting endogenous AS and perpetuating disease spreading to interconnected brain regions [51]. Thus, the most advanced clinical programs targeting AS include capturing extracellular AS with either passive immunotherapy, i.e., antibodies specific to AS or active immunotherapy, i.e., the administration of modified AS to induce the production of endogenous antibodies. Clinical trials using the passive immunotherapy approach include prasinuzemab (PRX002/ RO7046015; Roche, Prothena), which is in phase II (NCT03100149), the PASADENA trial for evaluation of MDS-UPDRS changes, after results demonstrated safety and proof of target engagement as a robust reduction in free serum AS and dose-dependent increases in the antibody in cerebrospinal fluid [52]. BIIB054 (cinpanemab, Neurimmune/Biogen) is also in phase II to assess safety and the pharmacokinetic/dynamic profile and both appear to be more specific for aggregated AS (NCT03318523) [53]. MEDI1341 (AstraZeneca, Takeda) is in Phase I for safety, tolerability and pharmacokinetic/dynamic profile (NCT03272165) and ABBV-0805 (AbbVie) is entering Phase I (March 2020) for safety and tolerability (NCT04127695) while Denali Therapeutics is pursuing an investigational new drug (IND) application for ATV:aSyn. With respect to active immunotherapies, Affitope (PD01A/PD03A, Affiris) has completed phase 1 trials for safety, tolerability, immunogenicity and clinical activity for PD as well as MSA (NCT02618941) and UB312 (United Neurosciences) for safety, tolerability and immunogenicity (NCT04075318). These therapies have great potential if indeed, by reducing extracellular AS, the load of hypothetically toxic AS aggregates and further propagation of AS to anatomically interconnected brain regions is diminished.

We anticipate this year that the results of several ongoing clinical trials will demonstrate which, if any, AS-targeted therapeutic strategies stand a chance of slowing or halting the progression of PD. Either way, it is vital that we continue to build on our knowledge of AS’s physiological role to better understand its role in PD pathogenesis, especially considering the consequences of reducing AS synthesis. Moreover, the presence of LB pathology does not necessarily prove AS’s toxic role and it is possible that AS aggregation may be a mere epiphenomenon or protective mechanism involving the sequestration of misfolded protein [54]. The inherent difficulty in elucidating the significance of LBs also highlights the importance of focusing efforts on identification of early biomarkers in the prodromal stage of disease to achieve the greatest future benefit from disease-modifying therapies. Finally, the ultimate effectiveness and range of therapeutic benefit of targeting different AS forms and conformational states remains to be known as the toxic AS species has not been established. The same applies to small antibodies that only target extracellular AS: will the achieved reduction in AS load be sufficient to slow or halt disease progression? These questions necessitate rigorous preclinical assessment in multiple in vivo models (both genetic and environmental) and direct measures of extracellular AS levels to help de-risk clinical trials, especially when generalizing results to idiopathic cases.

### 3.4. AS-Targeted Biomarkers

No biomarker to date has reached the desired sensitivity/specificity in order to be used in clinical practice and aid clinical trials. A major challenge in the biomarker field is that other neurological disorders (MSA, Alzheimer’s disease, etc.) share similar pathologies with PD and many of these protein changes overlap [55,56]. As a result, it is important to develop robust assays of high sensitivity and specificity for differential diagnosis. AS is present in different biofluids [57]. As it is a protein readily expressed in the brain, the most promising biofluid source is cerebrospinal fluid (CSF), which represents a snapshot of the central nervous system environment [57]. However, blood, saliva and peripheral tissues are also good candidates and may be preferred as more accessible and less invasive [58]. Pathology has mostly been associated with aggregate inclusions in LBs, where ~90% of the protein is Ser129 phosphorylated [59]. Thus, identification of any differences in the levels of these species and the pathways involved in disease is critical in order to develop biomarkers for the prognosis, diagnosis and monitoring of PD (see Summary Box 1).

Box 1 **Overview of AS-targeted biomarkers.**

*CSF*

▪ Most studies report that PD patients have lower levels of total AS compared to healthy controls [60,61] but increased levels [62] or absence of changes in the levels of total AS have also been reported [63].
▪ Early-diagnosed treatment-free PD patients have a decline in total AS levels which can successfully predict cognitive decline but not motor defects [64]. Medication does not contribute to this decline in total AS levels [62,65].
▪ PD patients are reported to have increased levels of non-normalised oligomeric AS [56,62] and a proportional relationship was observed regarding oligomeric/total AS ratio to motor symptom severity [62].
▪ The ratio of phosphorylated AS (pS129) to total AS is found elevated in PD patients compared to healthy controls [56] while a different longitudinal study reports a decrease of pS129 [62].

*Blood*

▪ The results from total [66], truncated and oligomeric AS forms [48,67] isolation from plasma/serum are generally inconclusive.
▪ Elevated phosphorylated AS forms remain stable as the disease progresses while total AS levels are shown to increase during the years [68].
▪ Exosomes of neuronal origin, circulating in the plasma of PD patients, show higher levels of total AS compared to healthy controls [69] correlating weakly with the severity of motor symptoms [69].

*Saliva*

▪ Total AS is lower and negatively correlated with the oligomeric form in PD patients compared to healthy controls [70].
▪ Findings of pS129/total AS ratio being higher in neuronal exosomes of PD patients versus control samples correlating with poor motor assessment [71], however, this was not reproducible in a larger cohort [70,72].
▪ PD patients show elevated oligomeric AS or oligomeric/total AS ratio versus control samples [70,71] not associated with disease severity [72].

*Gut mucosa*

▪ PD patients show higher levels of intestinal AS compared to healthy controls [73,74].
▪ Detection of AS conformational variants considered pathological is inadequate for diagnosis or prediction of PD [75].

### 3.5. CSF

Overall, there is a consensus that total AS levels are lower in CSF coming from PD patients compared to healthy controls [60,61]. This reduction may be explained by the degeneration of neurons/synaptic loss, increased inclusion of AS in LBs or by defects in protein’s gene expression, which are all hallmarks of the disease [61,63]. AS levels are not only lower in CSF but can also differentiate PD patients from healthy controls [76]. Nevertheless, no differences in AS levels have also been reported [63]. This discrepancy may be explained by the smaller cohort in the latter study, by differences in the antibody affinities used or by possible CSF contamination with AS from blood that can contribute to false-positive measurements [76]. On the other hand, total AS alone is likely insufficient for PD diagnosis, characterised by lack of specificity and, instead, total levels should be examined as a ratio to ubiquitously expressed control proteins [77]. Considering that toxicity linked to PD pathology is mostly associated with truncated forms of AS, current research has shifted attention to examining each of these species separately and in relation to total levels. Immunoprecipitation and mass spectrometry experiments have demonstrated the presence of these modified forms in CSF [78,79]. For example, despite the lower levels of total protein observed, pS129/total ratio appears to be elevated in PD [56], and the same is true for non-normalised oligomeric protein [55]. In this regard, different outcomes can arise depending on the type of species under investigation, thus, ideally, multiple biomarkers should be assessed simultaneously.

As PD is a progressive disorder, biomarkers for severity progression are needed. The correlation of protein levels with motor deficits, cognitive decline and disease severity has proven to date [55,80]. Some promising results were obtained from a longitudinal study monitoring patients for 2–8 years [64]. Total AS CSF levels were low in early-diagnosed, treatment-free PD, dictating that this change happens early in the disease and is irrespective of drug use [64]. It was also shown that total α-synuclein was diminished during the first 2 years and this could successfully predict cognitive decline but not motor defects [64]. Two cross-sectional studies in drug-naïve patients further confirmed that medication does not contribute to this decline in total α-synuclein levels [65,81]. On the contrary, in a second longitudinal study total levels as well as oligomeric AS increased whereas pS129 decreased over the 2-year course [62]. A proportional relationship was also observed regarding oligomeric/total AS ratio to motor symptom severity [62]. These controversial findings need further elucidation as to whether any of these species can be reliably used as a biomarker target in PD. Additionally, a gap in the experimental field is the lack of routine assays available to detect the aggregated protein form that is greatly implicated in pathology. Real-time quaking-induced conversion (RT-QuIC) is a new, promising assay providing an alternative way of detecting seeding species in CSF by amplification, characterised by very high specificity and sensitivity [82,83,84].

### 3.6. Blood

Although blood is an easily accessible biofluid source, results from AS isolation from plasma/serum are generally inconclusive for biomarker purposes [66]. According to an early study, levels of total AS are lower in PD patients compared to healthy controls and this is more apparent in early-onset versus late-onset PD [85]. A later study, however, showed elevated plasma AS positively correlating with cognitive decline [66]. Other groups failed to report such differences and more studies are needed to clarify these discrepancies in the literature [58,86]. Controversial findings also exist for the detection of truncated AS and oligomeric forms in plasma as well [49,67]. Regarding pS129 phosphorylated forms, elevated levels remained stable for up to 20 years after diagnosis and total levels were shown to increase during the years, asserting that total α-synuclein could potentially be used as a marker for disease progression [68].

Radiolabelled AS administration to mice brains has demonstrated that it can be readily transported to blood circulation, probably via exosome release through the blood brain barrier (BBB) [69,87]. Exosomes are small vesicles (~30–150 nm) of the endocytic pathway that can move from cells to the periphery, carrying the intracellular cargo into the body’s circulation [88]. Hence, it is believed that exosomes originating from the brain circulate in plasma, and upon isolation their content will be reflective of the neuronal state [87]. There are generally more consistent findings based on this approach rather than measuring α-synuclein freely floating in plasma; however, neuronal exosome isolation can be challenging. Early evidence came from immunoprecipitation studies against L1CAM neuronal exosome marker [69]; their presence was confirmed by Immuno-EM [69], however, only a small fraction of injected AS was enclosed in exosomes while the rest was detected as a free form in the plasma [69]. In contrast to lower levels of AS in CSF exosomes of PD patients, plasma exosomes had higher protein levels either alone (in exosomes only) or when normalised to total protein in whole plasma [69]. This increase in plasma may also explain the lower levels in CSF as a result of exosomal leakage to the periphery, probably serving as an alternative clearing mechanism of toxic species, since cell degradation pathways are defective in PD [89]. There was a weak correlation between high AS levels and severity of motor symptoms, while there was no such link with CSF samples, suggesting that plasma may be more useful as a biomarker for disease progression [69,90].

### 3.7. Saliva

The use of saliva in biomarker studies is a current trend as there is increasing evidence of AS being a major component [91,92]. From a recent pilot study, it was shown that exosomes isolated from saliva by ultracentrifugation are of a neuronal origin and express α-synuclein [71]. They demonstrated by immunoblotting that pS129/total α-synuclein ratio is higher in PD L1CAM-postitive exosomes versus control samples [71]. Disease progression could not be related to these high levels, attributed to the small number of participants, but there was a correlation with poor motor assessment [71]. In a larger cohort, however, total protein, pS129 or pS129/total ratio were stable, whereas oligomeric or oligomeric/total protein was elevated [70,72]. No association with disease severity and high oligomeric levels was found [72]. Total protein was lower and negatively correlated with the oligomeric form [70].

### 3.8. Gut Mucosa

AS measurements in the gastrointestinal tract (GIT) have also been proposed as a potential PD biomarker based mainly on the Braak hypothesis that states that the origins of PD pathology may begin in the GIT [93] and subsequently reaches the CNS via the dorsal motor nucleus of the vagus nerve [94]. However, despite the lack of complexity characterizing in vivo biopsies, the heterogeneity of the GI tract adds to the variability rising from different methodological approaches (i.e., antibodies, antigen retrieval methods, techniques) [75]. Transgenic animal models overexpressing AS show higher levels of intestinal AS [73,74] corroborating what was previously reported in PD patients [95,96]. However, despite earlier work delineating the use of GI AS as a predictive biomarker [95,96,97], more recently, the detection of pathological AS conformational variants has been considered inadequate for the diagnosis or prediction of PD, in spite of using different methods for optimal specificity and sensitivity [75]. Altogether, the detection of specific AS species (i.e., phosphorylated and oligomeric AS) when compared to total AS levels, and samples most representative of LB origin (i.e., neuronal-derived plasma exosomes and saliva), appear to be the most promising AS-related PD biomarkers.

## 4. Therapies Targeting LRRK2

Quite soon after the cloning and identification of the LRRK2 gene in the PARK8 locus on chromosome 12 [98,99,100], it was established that mutant forms of the protein that segregate with the disease could cause the death of CNS neurons [101]. Subsequent studies went further, and demonstrated, using both genetic and pharmacological approaches, that this neuronal death was kinase-dependent (e.g., [102,103]). While it remains to be demonstrated what the relevant substrate(s) are, and in which specific tissues (e.g., cell autonomous vs. non-cell autonomous) they are found, this kinase dependency was also demonstrated in viral in vivo models [104,105]. With these findings in hand, considerable resources, both private and academic, were dedicated to the search and characterization of potent and selective small molecule inhibitors of LRRK2 kinase activity, with the goal of identifying novel therapeutic candidates. First-generation LRRK2 kinase inhibitors (e.g., IN-1, CZC-25146; [106,107]) showed decent affinity and moderate selectivity, however even at lower concentrations still exhibited off-target effects and were unable to cross the blood-brain-barrier (BBB). Nevertheless, they served, and are still used, as useful “tool inhibitors” in in vitro and cellular studies of LRRK2 activity. Subsequent generations of inhibitors exhibited greatly improved potency, selectivity, and the ability to cross the BBB.

In 2012, Genentech, with support from the MJFF-LRRK2 Safety Initiative, published the findings of a pre-clinical safety trial of two structurally distinct LRRK2 kinase inhibitors, GNE-7915 and GNE-0877, in rodents and non-human primates [108]. Both compounds showed very high selectivity for LRRK2 among several kinase libraries, and exhibited a low nM-range IC50’s, in in vitro assays [108]. Both compounds are able to cross the BBB, and induced robust de-phosphorylation of pS935-LRRK2 (a marker of LRRK2 inhibition). Interestingly, while, in the brain, total LRRK2 levels were unaffected by 15 days of dosing with two doses of each compound, in the kidney, which expresses very high levels of LRRK2, total LRRK2 levels were significantly reduced [108]. This observation was markedly clear in the mouse, however, in the non-human primate model, brain levels of total LRRK2 trended lower as well. The explanation for this difference is unclear, however, it is noteworthy that a specific fragment of LRRK2, corresponding either to a translated splice variant or a proteolytic processing of full-length LRRK2, is observed selectively in kidney extracts. With relevance to their use in human clinical trials, however, exposure to both compounds induced an accumulation of lysosome-related lamellar bodies in type-II pneumocytes in non-human primates, phenocopying an observation in LRRK2-deficient mice [109]. Follow up safety studies in non-human primates indicated that the pathological changes observed in type II pneumocytes in treated animals were reversible and, importantly, did not lead to appreciable functional deficits in pulmonary function [110].

In 2018, Denali Therapeutics Inc. launched clinical trials of two distinct LRRK2 kinase inhibitors, DNL151 and DNL201, at centres in The Netherlands (NCT04056689) and the United States (NCT03710707), respectively. The first Phase I study was designed to assess safety, tolerability, pharmacokinetics, and pharmacodynamics in healthy volunteers using two doses of the DNL201 kinase inhibitor. In addition to evaluating the metabolism and stability of the compound, key outcome measures included biochemical indicators of target engagement, i.e., that LRRK2 kinase activity was in fact reduced in subjects receiving each dose of the test compound. We discuss the currently available LRRK2-targeted biomarkers in more detail below; however, in this clinical study, phosphorylation of LRRK2 at the Ser935 residue as well as phosphorylation of the LRRK2 substrate Rab10 at the Thr73 residue, was assessed in several biofluids (e.g., whole blood or PBMCs, CSF). Initial findings released by the company for both compounds indicated that targets for safety as well as target engagement were both met with both compounds. It is expected that clinical evaluation with one of the compounds will proceed to the next stage, with recruitment of PD patients both with and without the common G2019S mutation [111]. A potential obstacle might arise in finding adequate numbers of mutation carriers in order to have a sufficiently powered study to evaluate the efficacy of these compounds. This issue will potentially create a “bottleneck” as additional investigational compounds enter the playing field, competing for study participants. Nevertheless, the early, encouraging results of these compounds can only serve to embolden the approach of targeting LRRK2 kinase activity as a potential disease-modifying therapy.

Alternative approaches to block the kinase function of LRRK2 include ASOs, where the aim is to reduce the expression of LRRK2, thereby reducing the net kinase activity in the target cells. ASOs are single-stranded RNA oligomers that are complementary to specific sequences within a mRNA molecule. Once bound, the mRNA is digested typically via RNAse H. An example of such an approach is the compound BIIB094/ION859, developed jointly by Biogen and Ionis pharmaceuticals. There is an actively recruiting Phase I safety trial currently underway assessing single and multiple ascending doses (NCT03976349). In a recent collaborative study between Ionis Pharmaceuticals and researchers at the University of Alabama, LRRK2 ASO’s proved to be effective in reducing AS pathology in several models of iPD. First, in long-term cultures of primary hippocampal neurons, two independent ASO’s targeting LRRK2 effectively reduced the appearance of pS129-AS inclusions following treatment with AS pre-formed fibrils (PFFs; [112]). Extending this to an in vivo PFF model, the authors reported that both LRRK2 ASO’s reduced the abundance of pS129-AS inclusions by approximately 50% in comparison to controls [112]. There are several key findings to highlight from this work. First, this serves as another important piece of evidence indicating that CNS LRRK2 plays a critical role in PD progression, even in models of iPD, not associated with LRRK2 mutations. Secondly, it strengthens the link and further defines the pathway between LRRK2 activity and AS pathology in PD. In other words, as demonstrated in this model, pathological extracellular AS (PFFs) requires LRRK2 activity in order to induce downstream (i.e., in recipient neurons) pathological changes. Finally, this work demonstrates that centrally targeting LRRK2 activity appears to be sufficient in reducing AS pathology, although further work is needed to fully characterize, for example, cross-talk between CNS neurons and peripheral immune cell signalling. A further advantage of this approach is the inherent flexibility to modify the target sequence, thus providing the possibility to target only the mutant allele in heterozygous carriers of LRRK2 mutations. Such an approach has been taken in studies aiming to reduce the expression of mutant huntingtin protein [36] (for a summary of LRRK2 therapeutics, see Table 2).

One note of caution, pertaining to both approaches (small molecule inhibitors of LRRK2 kinase activity, or ASO down-regulation of LRRK2), concerns the uncertainties surrounding the long-term, system-wide inhibition of LRRK2 activity. It is unclear at this point whether the enduring inhibition of LRRK2 activity will negatively impact any vital functions of LRRK2 that are unrelated to its role in PD pathogenesis or progression. Moreover, while it has been clearly demonstrated that the inhibition of LRRK2 kinase activity is effective in pre-clinical models of familial and iPD, the relevant substrate(s) of LRRK2 linked to PD pathogenesis or progression have not been identified yet, highlighting the need for continued work in this area.

### 4.1. LRRK2-Targeted Biomarkers

A common underlying need in clinical studies evaluating LRRK2-based therapies is a quantitative, sensitive, and minimally invasive biomarker. Whether the specific outcome measure is reducing LRRK2 expression levels (e.g., the ASO approach), or reducing its kinase activity (e.g., small molecule inhibitors), reliable biochemical assays of well-defined targets are needed. Many biomarker studies focus on assessing LRRK2 posttranslational modifications and activity, as the pathogenic mutations have been associated with changes in protein phosphorylation levels and its endogenous substrates in in vitro and in vivo experiments [113,114]. If such changes prove to be distinct in biofluids of PD patients, they could potentially be used for drug target engagement and/or as inclusion/exclusion criteria in clinical trials of LRRK2 inhibitors. Lines of evidence highlight the central role of LRRK2 in the inflammatory response, which is robustly altered in PD [113,115,116]. As a result, considerable interest has arisen in mapping the protein’s expression patterns in different immune cell populations present in blood. Attention has also been directed to exosomal isolation from other peripheral biofluids such as CSF and urine, where LRRK2 (and other PD-linked proteins) is present [117,118,119,120] (see Summary Box 2).

Box 2 **Overview of LRRK2-targeted biomarkers.**

*Blood*

▪ LRRK2 is highly expressed in monocytes and neutrophils of PD patients [121,122]. Total LRRK2 levels are significantly elevated in CD16^+^ monocytes as well as T-cells and B-cells compared to healthy controls [121].
▪ Despite the inability to detect changes in total or phosphorylated LRRK2 levels in PBMCs of iPD patients [122,123,124], G2019S PD patients show reduced pS935 levels compared to iPD patients, G2019S healthy carriers and healthy controls, not correlating with motor symptoms or cognitive decline [125].
▪ The use of Rab GTPases is generally inconclusive. While the phosphorylation of Rab10 (pRab10) is reported to be LRRK2 dependent [126] there are no significant differences observed in PBMCs and neutrophils between PD patients and controls in pRab10 levels [122,127].

*CSF*

▪ No significant changes were observed between G2019S related PD patients and healthy groups in the levels of pS1292 [128].

*Urine*

▪ Normalisation of pS1292 levels to total LRRK2 from urinary exosomes can predict PD risk with different levels of accuracy among G2019S carriers with PD, PD non-carriers, sporadic PD patients and healthy participants of both sexes [117,118,119]. Poor clinical test scores are correlated with heightened pS1292 levels in male PD patients [119].
▪ Regardless of disease status, total LRRK2 levels are higher in females, though without any significant differences between PD and healthy groups [116,117,118,119].
▪ The levels of LRRK2 expression are higher in the PBMCs of males (14).

### 4.2. Blood

High expression levels of LRRK2 are evident in monocytes (particularly CD14^+^ and CD16^+^ types), which make up ~1%–20% of peripheral blood mononuclear cells (PBMCs), as well as neutrophils (another type of leukocytes) [121,122]. As shown by flow cytometry, total levels are significantly elevated in CD16^+^ monocytes, T-cells and B-cells in PD individuals compared to healthy controls [121]. In addition, this increase was positively correlated with cytokine release upon inflammatory stimulation in PD [121]. Phosphorylated levels of LRKK2 at pS910, pS935, pS955 and pS973 sites have also been studied in PBMCs [123,129]. pS910 and pS935 residues play an important role in modulating 14-3-3 chaperone binding [130]. Loss of this binding results in alterations in protein localisation and stability, whereas the role of pS955 and pS973 is currently unknown [129,131]. Initial studies have shown that pS910 and pS935 levels are not statistically different in PD, as measured by immunoblotting, suggesting an unlikely direct contribution of these modifications to PD pathology [123]. On the other hand, the application of specific LRRK2 kinase inhibitors ex vivo, such as PF06447475, has proven to be promising for drug treatment evaluations as they cause a dose-dependent loss of phosphorylation at these sites [129]. In this regard, pS910 and pS935 may not serve as reliable biomarker targets per se (i.e., as markers of disease progression); however, can be utilised in testing compound targeting in therapeutics. The inability to detect changes in total or phosphorylated LRRK2 levels in PBMCs raises questions as to whether there is a masking effect due to the heterogeneity of these cells; thus, the different subcellular types should be purified and examined separately for clarification in future experiments [122,123,124].

Upstream effectors and the pathways implicated in phosphorylating/dephosphorylating LRRK2 are largely unknown, with some likely candidates mentioned elsewhere [132]. While additional studies are needed, it has been suggested that the endogenous downstream LRRK2 substrates, Rab GTPases strongly involved in vesicular trafficking, can be used as a direct measure of kinase activity [133,134,135]. Phosphoproteomic screening identified numerous Rabs as substrates of LRRK2, among which Rab10 has attracted the greatest attention for biomarker studies [135]. An initial report showing that these proteins are expressed in PBMCs came from a small cohort study of 10 healthy donors [126]. Phosphorylated Rab10 (pRab10) was selectively attenuated upon treatment with Lu AF58786 and PFE 360 LRRK2 kinase inhibitors, indicating that this phosphorylation is LRRK2 dependent [126]. Levels of pRab12 were also examined; however, dephosphorylation did not correlate with LRRK2 inhibition [126]. Consistent results with previous findings for reduced pS935 levels were also shown, therefore, it was demonstrated that pRab10 quantification can potentially be used along with pS910 and pS935 in target engagement assays [126]. No significant pRab10 differences have been observed in PBMCs and neutrophils of PD patients and controls, implying that pRab10 may not be suitable as a PD biomarker per se in these cells, however, more work is needed with larger multi-centre cohorts [122,127]. MLi-2 and PF-06447475 inhibitors reduce pRab10 and pS935 in both PD and controls to a similar extent, whereas total levels remain unaffected, highlighting once again their potential use as peripheral drug targets [122,127]. Higher total LRRK2 levels have been observed in PD neutrophils versus controls, whereas this difference was not observed in PBMCs [127]. Of note, one disadvantage of using neutrophils is that purification may be contaminated with CD16^+^ monocytes, possibly contributing to discrepancies in interpretation of the findings [136].

Unexpectedly, pS935 levels were reduced in G2019S PD versus iPD patients, and when normalised to total LRRK2 this reduction was also apparent between G2019S PD individuals versus G2019S healthy carriers and healthy controls [125]. We know from previous studies in overexpression systems of this mutant that it does not typically alter phosphorylation at this site [123]. These results demonstrate that this specific mutation negatively regulates pS935 by likely unique mechanisms in these cells, and that the high levels of over-expression in experimental models may obscure small reductions in pS935-LRRK2 levels in G2019S-expressing cells. There was no indication of correlation between motor symptoms or cognitive decline and low pS935 levels [125]. This was one of a few experiments where ELISA was used instead of immunoblotting, suggesting that more sensitive assays can reveal changes that otherwise may be masked. Similar experiments should be carried out in larger cohorts to assess pS935 levels in relation to pRab10 readouts for comparison. Similar studies should also be conducted in carriers of other pathogenic mutations in LRRK2, in order to determine if similar trends are observed as for G2019S-LRRK2.

The most reliable way of measuring LRRK2 kinase activity directly is by quantifying the levels of auto-phosphorylation at pS1292 residue, as described in many experimental models (e.g., [114]). This is robustly detected in CSF and urinary exosomes, as described in the subsections below [117,119,128]. Peculiarly, no group has reported this modification in LRRK2 from PBMCs, and it is unknown whether phosphorylation at this site is detectable in LRRK2 purified from monocytes or neutrophils. This may mean that levels are below the detection limit of the assays currently available, or that this residue is not phosphorylated in these cells.

### 4.3. CSF

LRRK2 quantification with respect to CSF is mainly based on exosomal isolation rather than pure CSF itself. Isolation is achieved by various methods, as described elsewhere [137,138]. As mentioned previously in the AS section, monitoring changes in protein expression levels and activity in CSF originating exosomes can be used as a tool for PD biomarker studies. LRRK2-positive exosomes can emerge from various cell types in a mechanism that is 14-3-3 chaperone-dependent [118,139]. Levels of pS1292 have been detected in G2019S related PD and healthy groups, although no significant changes were observed between them [128]. The signal of pS1292 was highly saturated and any differences could have been underestimated [128]. By comparing these levels with corresponding pS1292 in urine exosomes (described in next the section) from the same participants, the signal was much more robust in CSF (~10-fold), whereas no correlation was found for total LRRK2 levels between the two biofluids [128]. What this possibly tells us about LRRK2 is that its activity is higher in the brain than in the periphery.

### 4.4. Urine

Urine is much more accessible and can be obtained at larger quantities than any other biofluid. Although the majority of exosomes in urine were thought to originate from the kidney, it is now evident from mass spectrometry and bioinformatics that there is a great diversity of the proteins present, coming from many tissues around the body, and PD-related proteins scored the highest according to PANTHER analysis [139]. However, as shown by cryo-EM, during the isolation process larger vesicles (>200 nm) can also be recovered, resulting in a heterogeneous population, potentially introducing an unknown variable [139]. Even so, LRRK2 is present in the lumen of urinary exosomes at detectable levels [98]. Intriguing findings came from a study where PD risk could be predicted by measuring pS1292 levels in a small cohort of male G2019S carriers with PD, which were ~5-fold higher compared to PD non-carriers when normalised to total LRRK2 [117]. Replication of these results in a larger cohort of male carriers with PD versus carriers without PD was successful, with ~3.6-fold elevation [118]. Most importantly, the same outcome was reproduced in urinary exosomes coming from iPD male and female patients versus healthy participants, with a ~1.5-fold difference [119]. Sex differences were also observed, where males had augmented pS1292 levels compared to females and were generally in correlation with poor clinical test scores [119]. This finding emphasizes once more the role of LRRK2 and its pS1292 modification as a potential therapeutic biomarker target in both iPD and G2019S-related PD cases. Furthermore, a relationship between auto-phosphorylation levels and medication was clear [119]. In another experiment, the contribution of sex was apparent, with total LRRK2 levels being higher in females, irrespective of disease status, but still without any significant differences between PD and healthy groups [117,119,120]. In PBMCs, however, there is an apparent reversal of the sex differences, with higher LRRK2 expression levels detected in males [14].

Lastly, evidence of Rab GTPase presence or phosphorylation in CSF or urinary exosomes has not yet been reported. A unique relationship between one specific Rab GTPase was recently described for Rab29, which is not only a substrate of LRRK2 but also an upstream activator. A proposed model demonstrated that both interact in a loop where Rab29 activates LRRK2, which then, in turn, phosphorylates Rab29 [140,141,142]. Whether Rab29 exist in human biofluids remains to be elucidated, and if it does, important findings may be disclosed due to its binary role.

## 5. Therapies Targeting GCase

Since their identification [23,143,144], heterozygous mutations in the gene encoding β-glucocerebrosidase (GCase), *GBA1* (GBA), are considered the greatest genetic risk factor for developing PD [9,145] and it is estimated that between 5%–25% of all PD patients carry GBA mutations [146]. Furthermore, PD patients that do not carry a GBA mutation demonstrate reduced GCase activity in the striatum and substantia nigra in conjunction with an accumulation of AS and alterations in lysosomal autophagy and lipid metabolism [147]. Phenotypically, iPD and GBA-PD are almost indistinguishable, except for an accelerated progression of motor and non-motor symptoms in GBA-PD patients [148,149]. GCase deficiency can lead to the accumulation of AS via the stabilization of oligomers, which in turn can cause a further decrease in GCase activity, leading to a bidirectional positive feedback loop effect [150]. This “toxic” relationship has been further advanced in vitro and in vivo and the prevailing hypothesis posits that GCase insufficiency leads to lysosomal dysfunction and subsequent proteinopathy in synucleinopathies [151]. Fascinatingly, GCase activity was found to be altered in LRRK2 patient blood samples [152], suggesting, along with the abovementioned data, an intriguing convergence of AS, LRRK2 and GBA.

GCase hydrolyzes glucosylceramide into ceramide and glucose. Homozygous or compound heterozygous *GBA1* mutations causes Gaucher disease (GD), a lysosomal storage disorder that leads to glycolipid accumulation in lysosomes. Although effective therapies are available for GD, including enzyme replacement therapy and substrate reduction therapy (i.e., glucosylceramide synthase inhibitors), they do not demonstrate target engagement in the CNS. Similar strategies currently in development for GBA-PD include boosting GCase activity via gene therapy, GCase small-molecule chaperones (SMCs) or glucosylceramide synthase inhibitors [153] (summarized in Table 3).

Gene therapy targeting the *GBA1* gene using an AAV-9 vector (PR001A) for use in GBA-PD is currently in Phase I, the PROPEL study, being tested for the safety of intra-cisternal administration in PD patients with at least one pathogenic *GBA1* mutation (Prevail Therapeutics; NCT04127578), while there are currently three ongoing phase II studies targeting GCase. The EMA-approved mucolytic, ambroxol, is an inhibitory SMC, i.e., it binds to the active site of GCase, inhibiting its activity, and it has demonstrated increased GCase activity in vivo along with a reduction in AS levels and target engagement in patients [154,155]. It is now being tested for repurposing in PD in the AiM-PD trial (University College London) for safety, tolerability and pharmacodynamic effects (NCT012941822) and in a separate trial for PD dementia (NCT02914366). LTI-291 is a non-inhibitory SMC (i.e., binds to a different site, not affecting activity) that is currently being tested in GBA-PD patients (Lysosomal Therapeutics; NTR7299). Venglustat (Ibiglustat, GZ667161/SAR402671) is a glucosylceramide synthase inhibitor, thus inhibiting the synthesis of glycosphingolipids. Venglustat is currently being assessed in the double-blind, placebo-controlled MOVES-PD study for efficacy and safety in patients with early-stage PD carrying a *GBA1* mutation (NCT02906020).

GBA-based therapeutic targets simultaneously underline the importance of patient stratification and sub-population specific clinical trials and the need for biomarker assays (i.e., GCase activity) for the assessment of target engagement and efficacy. If and when these trials demonstrate sufficient target engagement and efficacy, evidence suggests that such treatments may hold potential for use more broadly in idiopathic forms of PD.

### 5.1. GCase-Targeted Biomarkers

In most biomarker experiments that have being carried out in blood or CSF biofluids, GCase activity is generally lower in PD, irrespective of mutations. Based on the available findings, there is an indicative link between GCase, AS and LRRK2 PD pathology, as will be discussed here, and defining how the different pathways converge and interact in the disease is crucial for therapeutics. Hence, GCase activity has also been looked at in patients carrying mutations in AS or LRRK2 (see Summary Box 3).

Box 3 **Overview of GCase-targeted biomarkers.**

*Blood*

▪ GBA PD patients have lower GCase activity compared to non-carriers and healthy controls in dried blood spots [152].
▪ PD patients without the GBA mutation show a moderate decrease of GCase enzymatic activity [152] while lower activity has also been observed in polymorphic GBA variants though without significant change between iPD patients and healthy subjects [156].
▪ G2019S LRRK2 PD patients display higher GCase enzymatic activity [152].
▪ GBA mutation and polymorphic carriers have increased oligomeric AS species accumulated in plasma compared to iPD and healthy controls [157].
▪ There is defective GCase activity in the PBMCs of GBA patients versus A53T *SNCA* carriers or patients with iPD [156].
▪ GCase activity is diminished in monocytes of iPD patients [157].
▪ The higher levels of AS accumulation reportedly found in plasma exosomes of iPD patients versus healthy subjects are inversely proportional to GCase activity when normalised to free AS in plasma, with total AS levels in plasma remaining unaffected [158].

*CSF*

▪ Lower GCase activity, reported in GBA PD carriers and non-carriers versus healthy controls [159], is inversely related with higher total and oligomeric AS [160].

### 5.2. Blood

GCase activity was lower in GBA-PD patients versus non-carriers and healthy controls in dried blood spots and non-carriers alone were also characterized by a moderate decrease [152]. However, this group suggested that although it can contribute to pathogenesis, lower enzymatic activity is insufficient to cause PD; hence, it is not a reliable biomarker per se but can be considered as a tool to assess therapeutic target engagement [152]. In contrast, G2019S LRRK2 PD participants showed higher enzymatic activity compared with non-carriers and healthy controls, indicating that autophago–lysosomal pathways are differently regulated in this phenotype [152]. Lower activity has also been observed to a lesser extent in polymorphic *GBA1* variants; however, no significant change regarding iPD and healthy subjects was noted in this study [137]. Defective enzymatic activity was also present in PBMCs of GBA patients but not in A53T *SNCA* carriers or iPD [156]. Nevertheless, activity was diminished in the monocytes of iPD patients, a PBMC sub-population that expresses very high levels of GCase [127,158]. This difference can be attributed to a masking effect due to the heterogeneity of PBMCs.

As GBA mutations lead to loss of enzymatic function and autophago–lysosomal defects, it is speculated that AS cannot be sequestered for degradation effectively, building up in PD patient brains and this build up may further inhibit GCase activity [145,158]. This could explain the increased oligomeric species accumulated in plasma of *GBA* mutation and polymorphic carriers versus iPD and healthy controls as an attempt towards cell clearance [157]. Higher levels of AS accumulation have also been measured in plasma exosomes of iPD patients versus healthy subjects and this was inversely proportional to GCase activity when normalised to free AS in plasma [159]. On the other hand, total AS levels in plasma were unaffected [159]. This finding further highlights that enzymatic activity insufficiency is not only present in GBA PD but also in iPD. Additionally, studying protein levels in exosomes might be a more appropriate way to designate differences between health and disease rather than in free biofluids in general, where a variety of substances circulate, potentially masking any changes.

### 5.3. CSF

Fewer studies have been conducted in isolated CSF. Like in blood, there is lower GCase activity in CSF isolated from both GBA PD carriers and non-carriers when compared to healthy controls [160]. Furthermore, there is an established inverse relationship between GCase activity and AS levels, with lower activity, particularly early in the disease, and higher total and oligomeric AS [161]. As mentioned above, ambroxol is a promising repurposed compound that recently entered phase II clinical trials [155]. CSF from GBA PD patients demonstrated increased GCase levels and activity and an increase in AS concentration [155]. This may again imply an efflux-clearing mechanism of AS from the brain to the periphery, similar to that suggested in the blood [155].

## 6. Outstanding Issues and Questions

### Idiopathic PD: Is There Value in Targeting Genetic PD Players?

In addition to the loss of dopamine-producing neurons of the substantia nigra pars compacta, a key pathological hallmark of PD is the appearance of AS-positive cytoplasmic proteinaceous inclusions known as LBs. These structures have been detected in surviving DA neurons, and in most forms (idiopathic as well as familial) of PD. Notable exceptions to this include cases of familial PD associated with mutations in the *LRRK2* gene, where the neuropathology is heterogeneous [162]; although some pathology reports have suggested that LRRK2 can also be sequestered within LBs [163]. Thus, even in the absence of pathogenic mutations or multiplications in the *SNCA* gene, deposition of AS in surviving neurons is a common pathological finding in PD. While it is still unclear whether these dense proteinaceous structures are toxic, or simply discrete, confined, aggregates; the implication remains that AS plays a role in general, in PD. It has been postulated that the toxic conformation of AS is not the aggregated species detected in LBs, but rather soluble fibrils of the protein, which can also be transmitted trans-cellularly, possibly facilitating the progression of the disease. Thus, as in cases of familial PD attributed to mutations in the *SNCA* gene, therapeutic strategies targeting AS may be of benefit in iPD cases where the pathological involvement of AS also plays a role in disease pathogenesis or progression.

In addition to AS, another common autosomal dominant familial form of PD, as discussed above, is that associated with mutations in the *LRRK2* gene, located on chromosome 12 [99,100]. There are several lines of evidence implicating changes in LRRK2 activity in iPD as well. First, in 2016, the group of Andrew West published two studies [117,119] assessing changes in LRRK2 auto-phosphorylation (at Ser1292), as a proxy index of kinase activity, in urinary exosomes/extra-cellular vesicles (EVs). In the second of these two studies, the group reported that pS1292-LRRK2 levels were elevated in iPD, compared to healthy controls [119]. This was the first indication that LRRK2 activity is altered in forms of PD not associated with pathogenic mutations, which are known to increase kinase activity. Secondly, in an assessment of post-mortem tissue from iPD brain, Di Maio and colleagues applied the proximity ligation assay to demonstrate enhanced auto-phosphorylation of LRRK2 itself (S1292) and phosphorylation of its major substrate Rab10 (T73) as indices of LRRK2 kinase activation specifically in dopamine neurons in the ventral midbrain [164]. In addition, their findings of decreased pS129 AS levels and LRRK2 kinase activity in rotenone-administered rats, following LRRK2 kinase inhibition, also demonstrates the interplay between LRRK2 and AS and supports the potential use of LRRK2 inhibitiors in iPD. Finally, modulation of GCase activity is inversely correlated with AS levels in both GBA-PD and iPD and LRRK2 mutations also affect GCase levels, further demonstrating an interaction amongst the three gene targets. The reported milder phenotype of double *LRRK2* and *GBA1* variant carriers vs. GBA-PD further complicates this interrelationship but at the same time, provides additional evidence for bidirectional modulation and potential for broader use of specifically targeted therapeutics.

Overall, research centered on the underlying genetics, gene target convergence and their potential role in iPD should remain a top priority. Our growing knowledge of PD genetic architecture and the consensus that initially targeting specific subpopulations will lead to greater clinical success, has also created hope for a future of “precision neurology” for patients [165]. Even though we still have a lot to learn, armored with this constantly expanding knowledge base and innovative technologies, disease modification constitutes a more tangible goal than ever before. However, one of the many challenges hindering clinical trial success is the lack of biomarkers that can aid decision-making. Aside from the importance of environmental factors in PD, a new conceptual model consisting of triggers (agents that can initiate the disease process), facilitators (factors that aid disease spread in the CNS) and aggravators (elements that exacerbate pathology and spread disease beyond the basal ganglia), emphasizes the temporal importance of disease events [166] and therapeutic targeting (i.e., applying therapeutics when the target is most active); again, making evident the importance and urgency of biomarker development. Inevitably, a multifaceted approach to early/timely diagnosis, prognosis and treatment is required in complex, multifactorial and slowly progressing diseases such as PD and other neurodegenerative disorders. Perhaps we can learn a few lessons from multimodal models of diagnosis and treatment that have recently been proposed for vascular-related and mixed (vascular + degenerative) cognitive disorders [167,168] based on both pharmacological and non-pharmacological strategies. Adapted for PD, this first approach would include combinatorial drug therapy (with both existing and new disease-modifying drugs) targeting neuronal death and axonal loss, brain plasticity and compensatory mechanisms and protein misfolding in addition to non-pharmacological strategies such as transcranial brain stimulation and physical activity [167]. Similar to what we have proposed for PD, the second model highlights the usefulness of a panel of markers, including biofluids but also histopathological, imaging, and neurophysiological findings to enable prediction of “at risk” brains [168]. Altogether, translational medicine in PD research will benefit from clinical protocols based on multiple animal models (i.e., to maximally mimic the complete spectrum of disease) to improve patient stratification and the use of biomarkers in these models and in the clinic as outcome measures for disease-modifying therapeutic development and refinement.

## 7. Conclusions

As PD is a complex and multifactorial disease, the future research agenda must include a multidimensional approach involving individualized patient profiling and consideration of additional factors including sex and comorbidities. In this focused review, we have covered aspects of disease-modifying therapies relevant primarily to PD linked genetically to *SNCA*, *LRRK2*, and *GBA1* variants. While PD is a profoundly more complex disease than implied by this focus, there is ample evidence that each of the protein products of these genes may also play a role in multiple aspects of disease pathogenesis and/or progression, including mitochondrial dysfunction, oxidative stress, endoplasmic reticulum stress, impaired autophagy, axonal transport dysfunction, synaptic alterations and neuroinflammation, also affected in the more common idiopathic form of the disease. Further, it is apparent that a convergence exists between these proteins at the level of the lysosomal degradation system (e.g., [169]), providing further targets for novel therapeutic candidates as well as biomarker development—a critical component of drug discovery efforts. Along these lines, we have tried to emphasize, for each of the three targets, the need for validated, quantitative biomarkers that can track or predict disease progression, or serve as markers of target engagement in clinical trials of new therapies.

## Figures and Tables

**Table 1:** α-synuclein therapeutics.

Compound	Mechanism of Action	Clinical Indication	Patient Group	Sponsors	Clinical Phase	Time Period (Ongoing)	Clinical Trial
NPT200-11 (UCB0599)	Small molecule α-synuclein misfolding inhibitor	PD	Healthy volunteers	UCB, Neuropore Therapies	1: Safety, Tolerability, PK	07/2015–02/2016	NCT02606682
anle138b	α-synuclein aggregation inhibitor	PD	Healthy volunteers	MODAG GmbH Quotient Sciences Aptuit (Verona) Srl	1: Safety, tolerability, blood levels	12/2019	NCT04208152
Glyceryl phenylbutyrate *	α-synuclein clearance from brain to blood	PD	iPD	University of Colorado, Denver	1: Efficacy	01/2014	NCT02046434
Nilotinib *	Abl inhibitor; autophagy enhancer; boosts α-synuclein clearance	PD +/− dementia	PD +/− dementia iPD	Georgetown University Northwestern University	2: tolerability, safety	01/2017–10/2017	NCT02954978 NCT03205488
K0706 (SCC-138)	Abl tyrosine kinase inhibitor	PD	Clinically probable PD	Sun Pharma Advanced Research Company (SPARC)	2: safety, efficacy	02/2019-	PROSEEK NCT03655236
FB-101	c-Abl inhibitor; autophagy enhancer; boosts α-synuclein clearance	PD	Healthy volunteers	1^ST^ Biotherapeutics	1: safety, tolerability, PK	03/2019-	NCT04165837
Prasinezumab (PRX002/RO7046015)	α-synuclein passive immunotherapy	PD	iPD	Roche, Prothena	2: MDS-UPDRS changes	06/2017	PASADENA NCT03100149
BIIB054 (cinpanemab)	Human—derived antibodies; targets aggregated α-synuclein	PD	PD	Neurimmune, Biogen	2: Safety, PK/PD	01/2018	SPARK NCT03318523
MEDI1341	α-synuclein passive immunotherapy	PD	Healthy volunteers	AstraZeneca, Takeda	1: Safety, tolerability, PK/PD	10/2017	NCT03272165
ABBV-0805	α-synuclein passive immunotherapy	PD	iPD	AbbVie	1: Safety, tolerability	03/2020-	NCT04127695
ATV:aSyn	α-synuclein passive immunotherapy	PD	n/a	Denali Therapeutics	IND	-	-
Affitope PD01A/PD03A	α-synuclein active immunotherapy	PD	Early PD, iPD	Affiris	1: Immunogenicity, AEs	02/2016–02/2017	NCT01885494^ NCT01568099^ NCT02216188^ NCT02267434^ NCT02618941^
UB312	α-synuclein active immunotherapy	PD	Healthy volunteers	United Neuroscience	1: Safety, tolerability, immunogenicity	08/2019-	NCT04075318

Table 1 Alpha-synuclein (AS)-based disease-modifying treatment strategies and their current progress in clinical trials. iPD = idiopathic PD, * Repurposed drugs, ^ completed clinical trial, AEs = adverse effects, PK = pharmacokinetics, PD = pharmacodynamics; Clinical phases: 1 =Phase 1, clinical trials that focus on drug safety typically conducted with healthy volunteers to determine the drug’s pharmacokinetics and most frequent and serious adverse events; Phase 2, clinical trials that focus on the drug’s effectiveness in patients populations while continuing to study its safety and short-term adverse events.

**Table 2:** Leucine-rich repeat kinase 2 (LRRK2) therapeutics.

Compound	Mechanism of Action	Clinical Indication	Patient Group	Sponsors	Clinical Phase	Time Period (Ongoing)	Clinical Trial
DNL151 DNL201	Small molecule LRRK2 inhibitors	PD	PD PD, LRRK2-PD	Denali Therapeutics	1: Safety, tolerability, PK/PD 1b Safety, PD/PK	07/2019–12/2018	NCT04056689 NCT03710707
BIIB094	LRRK2 antisense oligonucleotides	PD	PD	Biogen, Ionis Pharmaceuticals	1: Safety, tolerability, PK	08/2019-	REASON NCT03976349

Table 2 Leucine-rich repeat kinase 2 (LRRK2)-based disease-modifying treatment strategies and their current progress in clinical trials. PK = pharmacokinetics, PD = pharmacodynamics; Clinical phases: 1 =Phase 1, clinical trials that focus on drug safety typically conducted with healthy volunteers to determine the drug’s pharmacokinetics and most frequent and serious adverse events; Phase 2, clinical trials that focus on the drug’s effectiveness in patients populations while continuing to study its safety and short-term adverse events.

**Table 3:** Glucocerebrosidase (GBA) therapeutics.

Compound	Mechanism of Action	Clinical Indication	Patient Group	Sponsors	Clinical Phase	Time Period (Ongoing)	Clinical Trial
PR001A	GBA1: PD-GBA AAV-9 gene therapy	GBA-PD	GBA-PD	Prevail therapeutics	1/2^^^: safety, tolerability, immunogenicity, biomarkers, efficacy	10/2019	PROPEL NCT04127578
Ambroxol *	Glucocerebrosidase chaperone: inhibitory SMC for GCase	Disease-modify PD PD dementia	PD PD + dementia	University College, London	2: Safety, tolerability, PD 2 Safety, tolerability, efficacy	12/2016–04/2018 (in press) 11/2015-	AiM-PD NCT02941822 NCT02914366
LTI-291	Non-inhibitory SMC	GBA-PD	GBA-PD	Lysosomal Therapeutics	2: Safety, tolerability, PK/PD	06/2018	NTR7299 (Netherlands Trial Register)
Venglustat (Ibiglustat; GZ667161/SAR402671)	Glucosylceramide synthase inhibitor	GBA-PD	GBA-PD	Sanofi Genzyme	2: Safety, tolerability, efficacy	12/2016	MOVES-PD NCT02906020

Table 3 Glucocerebrosidase (GBA)-based disease-modifying treatment strategies and their current progress in clinical trials. * Repurposed drugs, ^ first in-human study administering PR001A (gene therapy) in GBA-PD patients with a sham procedure as control; PK = pharmacokinetics, PD = pharmacodynamics; Clinical phases: 1 =Phase 1, clinical trials that focus on drug safety typically conducted with healthy volunteers to determine the drug’s pharmacokinetics and most frequent and serious adverse events; Phase 2, clinical trials that focus on the drug’s effectiveness in patients populations while continuing to study its safety and short-term adverse events.
